# Analytical Insights into Methods for Measuring Ischemia-Modified Albumin

**DOI:** 10.3390/molecules29194636

**Published:** 2024-09-29

**Authors:** Stefano Zoroddu, Angelo Zinellu, Ciriaco Carru, Salvatore Sotgia

**Affiliations:** Department of Biomedical Sciences, School of Medicine, University of Sassari, 07100 Sassari, Italy

**Keywords:** ischemia-modified albumin (IMA), albumin copper binding (ACuB) assay, albumin cobalt binding (ACB) test, acute coronary syndrome (ACS), biomarker

## Abstract

Ischemia-modified albumin (IMA) has emerged as a pivotal biomarker for the early detection of ischemic conditions, particularly myocardial ischemia, where timely diagnosis is crucial for effective intervention. This review provides an overview of the analytical methods for assessment of IMA, including Albumin Cobalt Binding (ACB), Albumin Copper Binding (ACuB), Enzyme-Linked Immunosorbent Assay (ELISA), new techniques such as liquid crystal biosensors (LCB), quantum dot coupled X-ray fluorescence spectroscopy (Q-XRF), mass spectrometry (MS), and electron paramagnetic resonance (EPR) spectroscopy. Each method was thoroughly examined for its analytical performance in terms of sensitivity, specificity, and feasibility. The ACB assay is the most readily implementable method in clinical laboratories for its cost-effectiveness and operational simplicity. On the other hand, the ACuB assay exhibits enhanced sensitivity and specificity, driven by the superior binding affinity of copper to IMA. Furthermore, nanoparticle-enhanced immunoassays and liquid crystal biosensors, while more resource-intensive, significantly improve the analytical sensitivity and specificity of IMA detection, enabling earlier and more accurate identification of ischemic events. Additionally, different biological matrices, such as serum, saliva, and urine, were reviewed to identify the most suitable for accurate measurements in clinical application. Although serum was considered the gold standard, non-invasive matrices such as saliva and urine are becoming increasingly feasible due to advances in technology. This review underscores the role of IMA in clinical diagnostics and suggests how advanced analytical techniques have the potential to significantly enhance patient outcomes in ischemic disease management.

## 1. Introduction

Ischemia-modified albumin (IMA) has gained attention as a potential point-of-care diagnostic marker for detecting ischemic events, particularly myocardial ischemia [1,2,3]. Traditional diagnostic tools for identifying cardiac injury, such as electrocardiography and cardiac biomarkers, including troponins, exhibit varied sensitivity windows, which makes early diagnosis challenging [3]. IMA is rapidly produced during ischemic episodes due to oxidative stress-induced modifications at the N-terminal region of albumin, which reduces its binding affinity for transition metals such as cobalt [4]. This specific alteration underpins various methods for measuring IMA concentrations in clinical practice [5,6].

The use of IMA has been explored in conditions beyond cardiovascular diseases, such as rheumatic diseases associated with chronic inflammation and recurrent ischemic episodes [7,8]. Elevated IMA concentrations observed in various rheumatic conditions, including rheumatoid arthritis and systemic lupus erythematosus, suggest its potential as a biomarker for ischemia-related changes in these diseases [8]. This broad applicability positions IMA as a valuable diagnostic tool not only for acute ischemic events but also for chronic inflammatory conditions [9].

Moreover, the relevance of IMA has also been explored in different non-cardiovascular and non-rheumatic conditions. Studies have shown that IMA concentrations are elevated in patients with chronic kidney disease (CKD) [10,11,12]. The underlying mechanisms are thought to involve pervasive oxidative stress and microvascular ischemia commonly observed in CKD, which positions IMA as a potential marker for monitoring disease progression and associated complications in these patients. Additionally, IMA has been examined in the context of sepsis, where it is believed to reflect the systemic hypoperfusion and microvascular dysfunction characteristic of this severe condition [13].

Increased concentrations of IMA in these patients have been associated with poorer outcomes, suggesting its potential as a prognostic marker in this critical condition. Furthermore, research on IMA has been extended to neurological disorders such as stroke and traumatic brain injury. The transport of albumin to the brain, particularly under ischemic conditions, may be compromised by disrupted blood flow, which could play a significant role in the pathophysiology of neurological disorders [14,15].

Higher IMA concentrations have been observed in patients with acute ischemic stroke compared to a control group of healthy individuals, and these elevated concentrations correlated with the severity of the event, serving as a measure of ischemic damage [16]. Similarly, in the context of traumatic brain injury (TBI), where secondary complications are often ischemic, IMA concentrations could provide valuable insights into the extent of brain injury and may serve as an indicator for guiding therapeutic interventions [17].

The clinical utility of IMA has been further extended to the early diagnosis and risk stratification of patients presenting with chest pain, where timely therapeutic intervention can significantly reduce morbidity and mortality [18,19,20]. These findings suggest that IMA could serve as a valuable biomarker not only for cardiac events but also for ischemia affecting other organs. Moreover, the integration of IMA measurements with other diagnostic modalities has the potential to enhance overall diagnostic accuracy and efficiency.

The detection of IMA can be achieved through various assays, including colorimetric methods such as the Albumin Cobalt Binding (ACB) test and the Albumin Copper Binding (ACuB) assay, immunoassays like ELISA, as well as more advanced techniques employing gold nanoparticles, liquid crystal biosensors (LCB), Quantum Dot-Coupled X-Ray Fluorescence Spectroscopy (Q-XRF), and mass spectrometry (MS). Each of these methods offers distinct advantages and limitations in terms of sensitivity, specificity, and feasibility for clinical application (Table 1). The ACB test is particularly prevalent due to its simplicity and cost-effectiveness; however, it is frequently subject to significant interference from other serum components [21].

This review aims to present a comprehensive analysis of the existing methodologies for IMA measurement, explaining their principles, analytical performance, and clinical relevance. The objective is to provide a clear understanding of the advantages and limitations of these methodologies in supporting appropriate clinical decision making. Additionally, recent advances in IMA-detection techniques and their integration with other biomarkers to develop new approaches for the early diagnosis and management of ischemic and inflammatory conditions are discussed.

## 2. Albumin vs. IMA

Human serum albumin (HSA) is a globular protein that serves as the primary carrier of various endogenous and exogenous compounds in the bloodstream, including hormones, fatty acids, and metal ions [22]. Structurally, HSA consists of 585 amino acids organized into three homologous domains (I, II, III), each consisting of two subdomains (A and B) [23]. The N-terminal region of albumin is of particular importance for its ability to bind divalent metal ions, such as copper (Cu²⁺), cobalt (Co²⁺), and nickel (Ni²⁺). In its native state, the first three amino acids at the N-terminus—aspartate, alanine, and histidine—form a high-affinity metal-binding site, playing a critical role in maintaining metal ion homeostasis and detoxifying free metal ions in the circulation [23].

**Figure 1:**
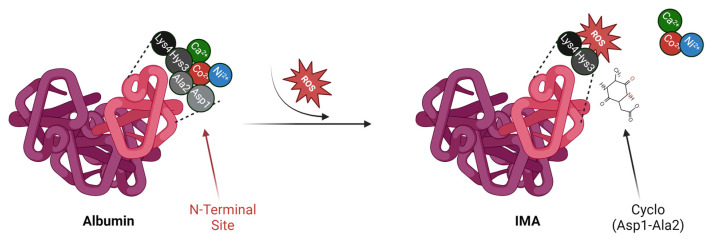
Schematic representation of IMA formation. Under normal physiological conditions, albumin binds transition metals such as calcium (Ca²⁺), cobalt (Co²⁺), and nickel (Ni²⁺) at its N-terminal region. During ischemic events, reactive oxygen species (ROS) induce structural changes in the N-terminal region, reducing its binding affinity and leading to the formation of ischemia-modified albumin (IMA). This is caused by the cleavage of the Ala2-His3 peptide bond through a nucleophilic attack by α-amino nitrogen on the carbonyl group, releasing a cyclic dipeptide (Asp1-Ala2 cycle). As a result, the truncated N-terminal sequence (NTS) is unable to bind transition metal ions, significantly decreasing the metal-binding capacity of albumin. Created with BioRender. Zoroddu, S. (2024) BioRender.com/f72b288.

During ischemic episodes, tissues are subjected to oxidative stress, a condition where the production of reactive oxygen species (ROS) exceeds the body’s antioxidant defense capacity [24]. This oxidative stress causes significant biochemical alterations to albumin, particularly at its N-terminal region. ROS interact with the metal-binding site, leading to structural modifications of the aspartate and histidine residues [25]. These oxidative changes result in a loss of albumin’s ability to effectively bind transition metals, giving rise to what is known as IMA. This mechanism involves the oxidation of the amino acids in the N-terminal sequence, altering the tertiary structure and reducing the availability of metal-binding groups, which diminishes albumin’s affinity for metal ions like cobalt. The structural modifications in IMA represent a breakdown in albumin’s physiological role. Normally, albumin acts as a scavenger for free metal ions, reducing the risk of metal-catalyzed oxidative reactions, such as those involving free copper, which can generate harmful hydroxyl radicals via the Fenton reaction. However, when albumin is modified to IMA, this protective function is impaired, leading to increased availability of unbound metal ions in the bloodstream, which can perpetuate oxidative stress and tissue damage (Figure 1). In this sense, IMA not only serves as a biomarker of ischemia but also reflects a disruption in the body’s metal homeostasis and redox balance. Physiologically, native albumin plays a key role in preventing oxidative damage by binding free metal ions, thus inhibiting the formation of ROS [26]. Its reversible binding to copper and cobalt is central to its detoxifying function. However, under ischemic conditions, where oxidative stress is elevated, the conversion of albumin to IMA represents a significant deviation from normal protein function. This transformation involves not only a loss of metal-binding capacity but also reflects oxidative damage that disrupts the protein’s overall structural integrity. Additionally, the altered conformation of IMA affects its ability to bind other ligands, potentially impacting the transport of fatty acids and drugs, further compounding the pathological state. Chemically, the difference between native albumin and IMA lies in the oxidative modification to specific amino acid residues, which leads to a reduction in the protein’s binding affinity for transition metals [27]. The N-terminal region of albumin is particularly susceptible to oxidative attack, as ROS can cleave or modify amino acid side chains, disrupting the coordination chemistry necessary for metal binding [26]. This conformational shift results in the reduced ability of IMA to chelate metals, thereby allowing free metal ions to contribute to further oxidative damage in the ischemic tissue. Pathologically, IMA serves as a sensitive and early biomarker of ischemia. Its rapid formation during oxidative stress, within minutes of ischemic insult, makes it a promising candidate for the early diagnosis of conditions such as myocardial ischemia. The increase in IMA concentrations in the bloodstream signals the onset of tissue damage well before irreversible injury occurs, providing clinicians with a critical diagnostic window to intervene [28]. The presence of IMA reflects not only the extent of oxidative stress but also a significant alteration in the functional capacity of albumin, transforming it from a protective transport protein into a marker of oxidative injury.

## 3. Analytical Methods for the Measurement of IMAs

### 3.1. The Albumin Cobalt Binding (ACB) Test

**Figure 2:**
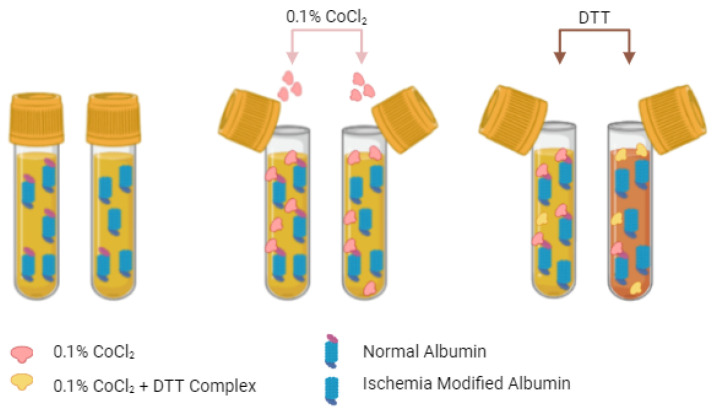
ACB Assay Procedure. A 200 μL serum sample is first mixed with 50 μL of CoCl_2_ (0.1%), followed by a 10-min incubation. Subsequently, 50 μL of dithiothreitol (DTT) is added, which binds to the free cobalt ions. The amount of free cobalt, which is inversely proportional to the concentration of IMA, determines the intensity of the resulting color. Higher IMA concentrations result in more free cobalt and greater color intensity.

The ACB test, a prominent colorimetric assay in ischemia diagnostics, exploits the distinct biochemical changes induced in albumin by ischemic conditions. This assay specifically measures the reduction in albumin’s affinity for cobalt ions (Co²⁺), which serves as an indicator of ischemic modification [1,18,21]. Bar-Or et al. were the first to develop an assay for IMA detection, pioneering the ACB test by elucidating the altered cobalt-binding properties of albumin in the context of ischemia and laying the groundwork for its clinical application [18]. In the ACB test, serum samples are treated with cobalt chloride, which binds to albumin (Figure 2).

Ischemic modifications to albumin decrease its affinity for cobalt due to structural alterations, resulting in a reduced capacity to bind Co²⁺ ions. When dithiothreitol (DTT) is added, it binds to the cobalt ions that remain free in the solution because of the diminished interaction with albumin, leading to the formation of a colored DTT-cobalt complex. This complex can be measured spectrophotometrically, and its intensity is inversely proportional to the loss of cobalt-binding capacity of albumin as a result of ischemic modification. Thus, the color intensity serves as a quantitative indicator of the extent of ischemic alteration of albumin, enabling a sensitive assessment of ischemic events in the clinical setting [18,19].

Since its introduction, the ACB test has been extensively researched to refine its accuracy, understand its limitations, and explore its potential integration with other diagnostic modalities to enhance the detection and management of ischemic conditions.

Bhagavan et al. [19] first evaluated the analytical performance of the ACB assay, demonstrating its effectiveness in detecting myocardial ischemia in clinical settings. The study found high sensitivity (88%) and specificity (94%) in distinguishing ischemic from non-ischemic conditions, highlighting the assay’s usefulness in emergency departments for the early detection of ischemia, potentially preventing irreversible myocardial damage. However, while effective in identifying myocardial ischemia, the ability of the ACB test to reliably distinguish between ischemic patients with and without myocardial infarction (MI) was limited.

Christenson et al. developed a modified ACB test to enhance its applicability for patients in intensive care units, undergoing surgery, receiving frequent blood transfusions, and patients on specific intravenous treatments where the use of anticoagulants, such as citrate or metal-chelating medications might commonly occur [29]. In this protocol, serum samples are pre-treated with calcium chloride (CaCl₂) to neutralize chelators commonly present in these samples, which can bind cobalt and interfere with the metal-binding assay. Once sample preparation is complete, the subsequent steps follow the standard ACB test protocol, as previously described.

The Beckman Coulter LX-20 and Cobas Mira Plus platforms have both been utilized to evaluate the analytical performance of the ACB test. Maguire et al. [30] demonstrated that despite robust indicators of precision (within-batch coefficients of variation (CV%) of 1.4, 2.0, and 2.5% at IMA concentrations of 88, 99, and 120 KU/L, respectively, and between-batch CV%s of 3.4, 3.3, and 3.0% at concentrations of 74, 84, and 123 KU/L, respectively), the measurements performed on Beckman Coulter LX-20 showed a negative bias of 7 KU/L compared to the Cobas Mira Plus, particularly at higher concentrations [31]. The reference levels established with the Beckman Coulter LX-20 platform were higher than the cut-off specified by the manufacturer using Cobas Mira Plus (110 vs. 85 KU/L). However, Zapico-Muñiz et al. [32], using Cobas Mira Plus, found that the 95th percentile concentration of IMA in their reference population of 101 KU/L was closer to the cut-off concentration reported by Maguire et al. [30] than that specified by the manufacturer. Additionally, several researchers have identified a range between 85 and 105 KU/L in IMA concentrations for acute coronary syndrome (ACS) patients with unclear clinical significance that requires further investigation [33,34]. These observations underscore the importance of re-evaluating and potentially standardizing cut-off values to ensure consistent and reliable diagnostic outcomes in clinical practice. Regardless of the platform used, variability in assay results depended on the presence of serum constituents such as bilirubin and fatty acids, which are known to compete with albumin for cobalt binding [35]. Moreover, the instability of serum IMA challenges its routine use, as samples must be analyzed within 2.5 h of withdrawal. Maguire et al. reported that serum IMA concentrations increased by 10% when samples were stored at 4 °C for prolonged periods. Conversely, storing samples at −20 °C preserves the stability of IMA without significant changes in concentration, even over extended periods [30]. Another critical factor to be considered for the effective use of the ACB test is the significant inverse relationship observed between total serum albumin and IMA concentrations. In this sense, van der Zee et al. [36] reported that IMA concentrations estimated in patients undergoing exercise myocardial perfusion scintigraphy were primarily reflective of serum albumin concentrations rather than indicative of myocardial ischemia. As proposed by Maguire et al. [30], this requires adjusting IMA values according to albumin concentrations to prevent potential misinterpretation of the results.

In summary, while the ACB test marks a significant advancement in the non-invasive detection of ischemia, its specificity and sensitivity, though adequate for initial screening, are subject to the limitations inherent to indirect biomarker-detection methods [37]. The assay does not directly measure ischemic modifications on the albumin molecule but rather infers these from altered cobalt binding dynamics, which may be influenced by non-ischemic factors affecting albumin’s binding properties [37]. Consequently, clinicians must interpret ACB test results within the broader context of patient symptoms and other diagnostic findings, such as an electrocardiogram (ECG) and troponin blood tests, as directed by the FDA [38]. Despite these analytical challenges, the ACB test’s role in the rapid screening for ischemic events remains valuable, particularly in resource-constrained settings where its ease of use and lack of need for sophisticated laboratory equipment make ischemia diagnostics more accessible. Furthermore, ongoing advancements in biochemical assays and a deeper understanding of ischemia’s molecular mechanisms promise to augment the sensitivity and specificity of IMA detection, potentially leading to the development of next-generation assays that offer enhanced diagnostic precision and clinical utility.

### 3.2. Cobalt vs. Copper

Traditional methods for detecting IMA primarily rely on the ACB assay, which exploits the reduced binding capacity of cobalt to the N-terminal region of albumin under ischemic conditions. Recent advancements, however, have introduced alternative metal-binding assays that offer potentially higher sensitivity and specificity. In particular, the ACuB assay has been developed as an innovative alternative to the ACB test [39]. The ACuB assay leverages the higher affinity of copper (Cu²⁺) for the N-terminal site of human serum albumin (HSA), which research has shown to be stronger than that of cobalt (Co²⁺). Eom et al. [39] demonstrated that Cu²⁺ binds more effectively to the N-terminal tetrapeptide of HSA, making it a more suitable candidate for detecting IMA.

The analytical performance of the ACuB assay is noteworthy. In controlled experiments, the ACuB assay was capable of detecting IMA in both normal and stroke-induced rat models with high sensitivity [4,39]. The assay clearly differentiated normal from ischemic conditions, with a significantly lower fluorescence signal in stroke models due to the increased presence of albumin-unbound free copper ions. These findings suggest that the ACuB assay could potentially offer more accurate detection of IMA concentrations compared to the ACB test, particularly in situations where cobalt may be less effective due to its weaker binding affinity. Moreover, the ACuB assay showed a strong linearity between copper-bound albumin and the IMA concentrations in serum samples, with a detection range that aligns well with the physiological concentration of albumin in human blood [39]. The optimized assay conditions, including the pH and incubation times, were carefully validated to ensure the reliability and reproducibility of the results. One of the significant advantages of the ACuB assay is its specificity. This study found that the fluorescence of Lucifer Yellow, a copper selective detecting agent, was effectively quenched by copper ions, with minimal interference from other metal ions such as nickel or iron, which could otherwise affect the assay results [39].

Finally, the ACuB assay represents a promising advancement in the detection of IMA, offering improved sensitivity, specificity, and overall diagnostic accuracy. With ongoing research, this method may become a valuable tool in clinical settings for the early detection of ischemic conditions, potentially outperforming the traditional ACB assay.

### 3.3. Liquid Crystal Biosensor (LCB)

The liquid crystal biosensor (LCB) is a new technology that combines the unique optical properties of liquid crystals with antibody-antigen interactions for the detection of IMA [20,40]. In the method outlined by He et al. [40], antibodies against IMA were immobilized onto a solid substrate, usually a glass slide. When a sample containing IMA interacts with the surface, its molecules are bound by antibodies, which disrupt the orientation of liquid crystal molecules in contact with the surface [40]. This disruption in liquid crystal optical properties is detected as a variation in light transmission or reflection, generating a measurable signal. The sensitivity of the LCB method to IMA is moderate, with the limit of detection measured at about 50 µg/mL [20]. While this provides adequate sensitivity for the detection of IMA, it may be insufficient for early stage ischemia when circulating concentrations of IMA are low. A significant factor in the specificity of LCB is the quality of the antibodies used. High-quality, specific antibodies ensure that the sensor primarily binds only IMA, minimizing false positives. However, cross-reactivity with similar proteins could reduce specificity. The LCB technique is relatively easy and inexpensive compared to more sophisticated techniques such as mass spectrometry. Because the instrumentation used in this assay is not complex, the method could be applied at the point-of-care or in resource-poor laboratories. The lower sensitivity of the method compared to other techniques may limit its clinical utility for detecting IMA at very low concentrations.

### 3.4. Quantum Dot-Coupled X-ray Fluorescence Spectroscopy (Q-XRF)

Quantum Dot-Coupled X-Ray Fluorescence Spectroscopy (Q-XRF) is an advanced method that combines quantum dots (QDs) with X-ray fluorescence to detect IMA [20,41,42]. Quantum dots are nanometer-sized semiconductor particles characterized by unique optical properties, such as intense fluorescence. In the Q-XRF method developed by Luo et al., quantum dots are conjugated to antibodies specific to IMA [42]. When a sample is introduced, IMA molecules bind to these quantum dot-conjugated antibodies [43]. Subsequently, IMA is detected by measuring the X-ray fluorescence emitted by the quantum dots after excitation with X-rays [20,42].

The fluorescence intensity is directly proportional to the concentration of IMA in the sample. Q-XRF is among the most sensitive techniques available, with a detection limit as low as 0.05 U/mL, making it one of the most precise methods developed for IMA detection to date [20,42]. This high sensitivity allows for the detection of very low concentrations of IMA, which is crucial for the early diagnosis of ischemic conditions. The specificity of Q-XRF is also high, primarily due to accurate targeting by quantum dot-conjugated antibodies, enabling the differentiation of IMA from other serum proteins with minimal interference. However, despite its high sensitivity and specificity, the practical application of Q-XRF in routine clinical settings is limited by the high cost of equipment and the need for specialized training. Thus, while Q-XRF represents a powerful tool for research and specialized diagnostics, its widespread clinical use is constrained by these challenges.

### 3.5. Mass Spectrometry (MS)

Mass spectrometry (MS) is a powerful analytical technique used to identify and quantify molecules based on their mass-to-charge ratio. For IMA detection, MS involves ionizing serum proteins, including albumin, followed by fragmenting these ions within a mass spectrometer [4]. The resulting fragments are then separated and detected based on their mass-to-charge ratios. MS can directly identify specific post-translational modifications of albumin indicative of ischemic changes, such as oxidation or truncation of the N-terminal region. This technique offers extremely high sensitivity and can detect even minute alterations in the molecular structure of albumin that occur during ischemia. As a result, MS is one of the most accurate methods for identifying IMA. The specificity of MS is excellent because it directly measures the mass and structure of molecules, allowing it to distinguish between IMA and other forms of albumin with high precision. This includes identifying specific oxidative modifications or truncations characteristic of ischemic conditions. However, despite its unparalleled sensitivity and specificity, the feasibility of MS in routine clinical practice is limited. The equipment is expensive and requires significant technical expertise to operate, and sample preparation is complex and time-consuming. As a result, MS is typically reserved for research applications or specialized diagnostic laboratories rather than for routine IMA screening.

### 3.6. Immunoassays: ELISA and Gold Nanoparticles 

Immunoassays, particularly ELISA, offer an alternative approach to estimating IMA by utilizing antibodies that specifically recognize ischemia-induced modifications in albumin [20]. Recent advancements in immunoassay techniques have led to the development of highly sensitive and specific assays capable of detecting subtle changes in IMA concentrations. Key improvements include the use of monoclonal antibodies with enhanced affinity for IMA and the use of advanced electrochemiluminescence detection systems, which significantly enhance the assay’s sensitivity and dynamic range. Despite these technological advancements, particularly in their application to routine clinical practice, significant challenges remain. Immunoassays are costly, require specialized laboratory equipment, and exhibit considerable variability among test kits.

Wu et al. [21] were the first to quantify IMA using ELISA, marking significant progress in the methodology for detecting IMA. ELISA measures the fraction of albumin that has lost its metal-binding ability due to ischemic conditions [21]. This assay captures IMA on a plate coated with specific anti-IMA antibodies, followed by detection with a secondary antibody conjugated to a reporting enzyme. The signal generated by the enzyme-substrate reaction is directly proportional to the IMA concentration in the sample.

The sensitivity of the ELISA method was reported as 39.1% at first presentation and improved to 55.9% with the addition of cardiac troponin I (cTnI), suggesting that their combination significantly enhances early diagnostic ability for acute myocardial infarction (AMI) [21]. Although lesion specificity was very good with ELISA alone, it decreased when combined with cTnI, possibly reflecting a compromise between sensitivity and specificity in identifying ischemic events that may not yet be associated with irreversible myocardial necrosis. Recent studies have further explored the application of ELISA in detecting IMA under various pathological conditions. For example, in a study by Ahn et al. [43], IMA concentrations were evaluated in patients with ANCA-associated vasculitis (AAV). Although the study did not find a significant correlation between IMA concentrations and disease activity in AAV, it highlighted the potential of ELISA to measure IMA in different clinical contexts, even outside cardiovascular diseases. In another study, Shaker et al. [44] evaluated the potential use of ELISA to measure IMA concentrations in patients with inflammatory diseases, further emphasizing its versatility as an assay applicable to various pathological conditions.

In addition to ELISA, gold nanoparticles (AuNPs) have also been used in immunoassays to enhance the sensitivity of detection techniques. Li et al. [45] developed a gold nanoparticle immunosensor enhanced by surface plasmon resonance (SPR) for the detection of IMA. This technique utilizes the optical properties of SPR, which are sensitive to refractive index changes on a gold surface. Compared to conventional SPR methods with a limit of detection (LOD) for IMA of 100 ng/L, 10 nm AuNPs achieved a LOD of 10 ng/L [45]. This represents a significant increase in sensitivity, potentially enabling the detection of IMA at much lower concentrations, which is crucial for early detection in ischemic conditions. In the study by Li et al. [45], the AuNP-enhanced SPR method also demonstrated high specificity, exhibiting less interference from heparin, hemoglobin, bilirubin, and triglycerides, which could otherwise cause false positives in other assays. These findings suggest that this method may overcome some of the limitations of existing IMA detection techniques, such as the ACB test, which is less reliable in patients with low serum albumin concentrations [45]. In terms of analytical performance, the SPR immunosensor with AuNP enhancement showed a signal enhancement ratio of 9.4-fold compared to the direct binding SPR assay without nanoparticles [45]. The precision and reproducibility of this method were confirmed through multiple measurements, underscoring its robustness for potential clinical application.

In conclusion, the integration of gold nanoparticles in SPR-based immunoassays offers a powerful tool for the highly sensitive detection of IMA, enhancing traditional methods like ELISA. These advancements not only significantly improve the analytical sensitivity and specificity of IMA detection but also broaden the potential clinical applications of these biomarkers in diagnosing acute coronary syndrome and other ischemic conditions.

### 3.7. Electron Paramagnetic Resonance (EPR) Spectroscopy

Electron paramagnetic resonance (EPR) spectroscopy is an advanced analytical technique that has been explored for detecting IMA by analyzing changes in the albumin molecule and its ability to bind metal ions [46,47]. EPR works by detecting unpaired electrons in paramagnetic substances, such as metal ions, which can bind to specific sites on albumin. In the laboratory, blood or serum samples are prepared either by introducing paramagnetic probes or relying on naturally occurring paramagnetic metal ions bound to albumin [47,48]. 

These probes help in detecting conformational changes in albumin’s structure. The sample is placed in an EPR spectrometer, where it is exposed to a magnetic field. Microwave radiation is then applied, causing the unpaired electrons in the paramagnetic centers to transition between energy levels, producing a resonance signal. This signal is highly sensitive to the molecular environment surrounding the metal ions, allowing researchers to detect changes in the albumin structure and its metal-binding behavior. The EPR spectrum provides detailed information about the strength of the interaction between albumin and the metal ions, revealing how ischemia has affected the albumin molecule [46,47,48]. 

Alterations in the resonance signal can indicate a reduction in metal-binding affinity, and the shape of the spectrum can reflect structural changes in the albumin molecule. These data make EPR spectroscopy particularly valuable for studying ischemic modifications to albumin and understanding the molecular underlying oxidative stress and ischemia. EPR spectroscopy is a powerful technique with high sensitivity and specificity, making it useful for detecting early signs of ischemia [49,50]. Its ability to detect changes at the molecular level allows for detailed analysis of albumin’s metal-binding properties, which can be critical in research related to ischemia, oxidative stress, and associated conditions. Additionally, because EPR can directly detect free radicals, it is especially useful for studying the role of ROS in ischemic conditions, such as in sepsis, cancer, and toxemia, where oxidative stress plays a significant role. Despite its advantages, EPR spectroscopy is complex and expensive, requiring specialized equipment and expertise to operate and interpret the results. This limits its accessibility in routine clinical settings, where simpler assays, such as the ACB test, are often preferred. While EPR provides detailed insights into albumin’s structure and function, its clinical application remains more limited due to these practical constraints. However, as a research tool, it offers unmatched specificity in analyzing albumin’s transport function and detecting structural changes resulting from ischemic events, contributing to a deeper understanding of ischemia and its biomarkers, such as IMA.

**Table 1:** Comparative overview of the detection methods.

Method.	Sensitivity	Specificity	Advantages	Litimations
Albumin Cobalt Binding (ACB)	70–85	75–90	Cost-effective, easy to use	Interference by bilirubin, fatty acids, moderate specificity
Albumin Copper Binding (ACuB)	85–90	90–95	Higher sensitivity and specificity than ACB, broader clinical use	More expensive, requires copper reagents
ELISA	40–60	55–75	Good for large-scale screening	Lower sensitivity compared to newer methods, variability
Liquid Crystal Biosensors (LCB)	Moderate	Moderate	Inexpensive and easy to use, potential for point of care	Moderate sensitivity, early in development
Quantum Dot Coupled X-Ray Fluorescence (Q-XRF)	High	High	Very high sensitivity, suitable for small sample volumes	High cost, complex equipment
Mass Spectrometry (MS)	Very high	Very high	Gold standard for specificity, highly accurate	Requires specialized equipment and expertise, high cost
Electron Paramagnetic Resonance (EPR)	High	High	High sensitivity for detecting transport and molecular changes	Limited availability, requires technical expertise

## 4. Comparative Analysis in Different Biological Samples

### 4.1. Serum

Serum, saliva, and urine are biological fluids in which IMA can be measured, each offering distinct advantages and limitations summarized in Table 2. Serum is the most conventional and widely used matrix for measuring IMA, providing the advantage of well-established reference intervals and extensive validation through numerous clinical trials. Serum-based IMA measurements are thoroughly documented and considered reliable, particularly for diagnosing acute coronary syndrome (ACS) [51]. However, several factors can affect the sensitivity and specificity of IMA assays in serum samples:

1. Preanalytical Variability: sample handling, particularly the time from collection to processing, can significantly impact IMA concentrations. Delays in processing or storage at temperatures different from those used during analysis may alter or degrade albumin, consequently affecting its metal-binding capacity and thus influencing IMA readings [52]. Specifically, IMA concentrations have been reported to remain stable for up to 24 h when serum is stored at 4 °C; however, significant degradation can occur with prolonged storage at room temperature [4].
2. Storage Conditions: the temperature at which serum samples are stored is critical for preserving the integrity of IMA [52,53,54]. Samples should be stored at –20 °C or lower if not analyzed immediately. Research indicates that –80 °C is the optimal storage temperature for maintaining IMA concentrations over extended periods [52]. At this temperature, the risk of protein degradation or modification is minimized, thereby reducing assay interference to a minimum [55]. While –20 °C is adequate for short-term storage, it may not be sufficient for long-term preservation, whereas –80 °C provides maximum stability.
3. Freeze–Thaw Cycles: IMA measurements in serum are significantly affected by repeated freeze–thaw cycles. Each cycle can lead to protein denaturation and a potential loss of albumin’s metal-binding sites, thereby reducing the accuracy of IMA detection [56]. To minimize this risk, it is recommended to aliquot serum samples before freezing, thereby avoiding multiple freeze–thaw cycles.

### 4.2. Saliva

Saliva offers a non-invasive alternative for measuring IMA and represents a growing area of research [57]. Sample collection is straightforward, requires no special instrumentation, and the risk of contamination is minimal; thus, it holds significant potential for point-of-care testing and large-scale epidemiological studies. However, several challenges and considerations must be addressed:

1. Assay Sensitivity: the concentration of albumin in saliva is significantly lower than in serum, presenting a challenge for detecting IMA with high sensitivity [58]. Existing assays require further optimization, potentially through the development of more advanced signal-detection methods or amplification techniques, to accurately measure IMA concentrations in saliva.
2. Influence of pH and Composition: the pH of saliva can vary significantly under different conditions, such as food intake, oral hygiene, and time of day [59]. These pH fluctuations may affect the stability of IMA and, consequently, the efficiency of the detection test, leading to variability in results [60]. Additionally, interference from other proteins, enzymes, and contaminants in saliva could affect the measurement of IMA, necessitating the development of robust sample-preparation protocols to mitigate these effects [59,60].
3. Temperature and Stability: although the stability of IMA in saliva under different storage conditions has not been studied as extensively as in serum, it is known that protein biomarkers in saliva are sensitive to storage temperature. Therefore, freezing at -80°C is recommended for optimal preservation, as suggested by practices used for other protein biomarkers in saliva [61]. However, further research is required to determine the most effective storage conditions for IMA in saliva to ensure its long-term stability.

### 4.3. Urine

Urine is another non-invasive matrix under exploration for IMA measurement, offering the potential for continuous monitoring of ischemic conditions and non-invasive assessment of treatment efficacy. However, urine presents unique challenges:

1. Albumin Concentration: the lower concentration of albumin in urine compared to serum or saliva makes detecting IMA more challenging [62]. Therefore, assays must be highly sensitive and specific, potentially requiring concentration steps or the development of enhanced detection methodologies to accurately quantify IMA in urine samples.
2. Influence of Urine pH and Composition: the pH and composition of urine can vary depending on factors such as hydration status, diet, and kidney function [63,64]. While these variations are known to affect the stability and binding properties of other proteins in urine, their specific impact on IMA detection remains unclear and warrants further investigation. Therefore, it is important to account for these variables when developing and standardizing urine collection and preparation protocols to minimize potential variability in assay results.
3. Storage Conditions: similar to other biological fluids, urine samples may be susceptible to degradation when stored at room temperature [65]. The stability of IMA in urine under different storage conditions has not been extensively studied, requiring further research to establish optimal preservation methods. Although it is generally accepted that lower storage temperatures, such as −80 °C, may prevent the breakdown of proteins and other biological molecules, including IMA, this recommendation is based on general best practices for protein preservation rather than specific evidence for IMA in urine [65]. Therefore, further studies are needed to determine the most effective storage conditions for maintaining the integrity of IMA in urine samples, particularly for long-term storage.

**Table 2:** Overview of biological matrices.

Biological Matrix	Advantages	Limitations
Serum	Gold standard, reliable, well-validated in clinical settings	May require rapid processing for accurate results
Saliva	Non-invasive, easy to collect, promising with technological advancements	Lower albumin concentration, assay sensitivity challenges, variable pH
Urine	Non-invasive, potential for continuous monitoring, easy to collect	Lower albumin concentration, assay sensitivity challenges, variable pH and composition

### 4.4. Other Factors Affecting Sensitivity and Specificity

In addition to matrix type and storage conditions, several other parameters can influence the sensitivity and specificity of IMA detection:

1. Interfering Substances: the presence of other proteins, metal ions, or contaminants in the sample may compete with or interfere with IMA detection [42,66]. For instance, high levels of free copper or cobalt ions could falsely alter IMA readings by affecting the metal-binding sites on albumin.
2. Assay Calibration: the calibration of assays using appropriate standards is critical for maintaining accuracy. Variability in the calibration process between different laboratories or analyzer models can lead to variability in results [67].
3. Timing of Sample Collection: the timing of sample collection relative to the onset of ischemia can significantly impact IMA concentrations. Since IMA concentrations peak within a few hours after ischemia onset and then decline, the timing of sample collection can affect detection sensitivity [68].

Although serum remains the gold standard for IMA detection, saliva and urine offer promising non-invasive alternatives. The success of IMA detection across these different matrices, however, depends on optimizing assay sensitivity, ensuring proper sample handling and storage, and minimizing preanalytical and analytical variability.

## 5. Conclusion

This review provides a comprehensive overview of the methodologies and matrices used for IMA detection, focusing on the analytical performance, advantages, and limitations of each approach. Through a critical analysis of the current literature and a comparative assessment of different detection methods in various biological samples, this review highlights the clinical relevance and potential of IMA as a biomarker for early ischemic event detection, particularly in ACS. The clinical significance of IMA evaluation is considerable, as IMA concentrations rise within minutes of ischemia onset [69], offering a critical diagnostic window before irreversible myocardial damage occurs.

Among the methods reviewed, the ACuB assay emerges as particularly promising [39], offering higher sensitivity and specificity compared to the traditional ACB test due to the stronger binding affinity of copper to the N-terminal site of albumin [27]. The ability of the ACuB assay to differentiate IMA concentrations in various ischemic conditions, such as myocardial infarction and stroke, underscores its potential for broader clinical applications. Additionally, the assay’s robustness across different biological matrices, including serum, saliva, and urine, positions it as a versatile tool for IMA detection.

The review also discusses other advanced methods for IMA detection, such as LCB, Q-XRF, MS, and EPR. The LCB assay provides a simple, cost-effective option with moderate sensitivity, though it may be less effective for detecting low IMA concentrations. Q-XRF offers remarkable sensitivity and specificity, but its complexity and cost limit widespread use. MS, known for its accuracy in identifying specific ischemic modifications to albumin, is the gold standard for sensitivity and specificity, though its high cost and technical demands restrict it to specialized settings. EPR spectroscopy offers high specificity in detecting changes to albumin’s transport function and binding properties due to ischemia-induced oxidative stress, making it a highly informative research tool. However, its technical complexity and cost limit its broader clinical application despite its high sensitivity and utility in analyzing IMA. Regarding biological matrices, serum remains the gold standard for IMA measurement, providing reliable and extensively validated results. However, non-invasive alternatives such as saliva and urine show promise, especially with advancements in assay sensitivity and specificity. 

For instance, although IMA concentrations in urine have been less thoroughly characterized compared to serum, studies suggest that urinary IMA levels are directly correlated with renal ischemic conditions and kidney dysfunction, which affect the albumin-filtration process. As a result, the clinical utility of urine as a matrix for IMA detection remains under investigation, and further research is needed to establish standardized reference ranges and detection limits. The development of highly sensitive automated analyzers and novel detection techniques further enhances the potential of these non-invasive matrices in clinical practice.

In conclusion, IMA measurement is a powerful diagnostic tool with the potential to significantly improve clinical outcomes in patients with ischemic conditions. Future research should focus on refining detection methods, standardizing protocols, and exploring the full clinical utility of IMA in various ischemic diseases. As the field progresses, integrating advanced detection techniques, such as the ACuB assay, into routine clinical practice could facilitate earlier and more accurate diagnoses, ultimately improving patient care and outcomes.

## Data Availability

Not applicable.
